# High-intensity interval training in breast cancer survivors: a systematic review

**DOI:** 10.1186/s12885-021-07804-w

**Published:** 2021-02-22

**Authors:** Katsunori Tsuji, Yutaka J. Matsuoka, Eisuke Ochi

**Affiliations:** 1 Division of Health Care Research, Center for Public Health Sciences, National Cancer Center Japan, Tokyo, Japan; 2 Faculty of Bioscience and Applied Chemistry, Hosei University, Tokyo, Japan

**Keywords:** Cancer survivor, High intensity interval exercise, Endurance performance, Home-based exercise

## Abstract

**Background:**

To review the settings and outcomes of high-intensity interval training (HIIT) interventions for breast cancer survivors, and to explore the feasibility of prescribing exercise for breast cancer survivors.

**Methods:**

A systematic search of electronic databases was conducted for studies published up to May 31, 2020. Eligibility criteria included randomized controlled trials of HIIT intervention in breast cancer survivors. Studies were grouped by whether the intervention was conducted during or after breast cancer treatment, and intervention methods and outcomes were reviewed within each group.

**Results:**

Twenty-six studies were identified, and 13 satisfied the inclusion criteria. Intervention was conducted during treatment in 8 studies, and after treatment in 5. Intervention duration ranged from 3 to 16 weeks, with 2 or 3 sessions per week, for a total of 9 to 36 sessions. All interventions were supervised; 12 were lab-based, and 1 was community-based. One of most promising outcomes was improvement of cardiorespiratory fitness by HIIT.

**Conclusion:**

This review found that all studies on HIIT for breast cancer survivors investigated lab-based, supervised interventions, but not home-based or unsupervised. HIIT is a time-efficient method for increasing cardiovascular function in breast cancer survivors, but further research is necessary to determine its effects on other outcomes.

## Background

Breast cancer survivors are suffering many problems with the complications of surgery and radiation therapy such as Axillary web syndrome, pain, limited range of motion and dysfunction of the upper limbs, posture imbalance, lymphedema, or psychological symptoms associated with those conditions. The rehabilitation approach is effective for these sequelas [1–7]. The strong association between physical activity and all-cause mortality risk in breast cancer survivors [8] has led experts to recommend that breast cancer survivors engage in physical activity and exercise [9] and prompted researchers to investigate exercise interventions for this population. Combination of aerobic training and resistance training is considered particularly effective [5, 9].

High-intensity interval training (HIIT) interventions have recently been proposed as a promising method for quickly improving fitness. HIIT consists of repeated sets of short bursts of high-intensity exercise followed by a rest interval, and has been shown improve fitness in both athletes and the general population [10, 11]. In recent years, research on the suitability of HIIT for cancer survivors has emerged as well. Systematic reviews and meta-analyses of HIIT for cardiorespiratory fitness in cancer survivors have already shown HIIT to have some degree of effectiveness [12, 13]. Research on HIIT for breast cancer survivors was first published around 2016 [14], but no review article focusing exclusively on breast cancer survivors has been published to date.

Therefore, the purpose of this review is to determine whether there is a home-based HIIT intervention in breast cancer survivors. The specific characteristics of interest were (1) timing (during or after treatment), (2) setting (lab-based, community-based, or home-based), and (3) supervision (supervised or unsupervised). we thought that to devide the intervention timing is important to consider the rehabilitation approach (setting, exercise supervision etc..) of breast cancer survivors. In addition, in light of concerns that sheltering in place during the novel coronavirus (SARS-CoV-2) pandemic of 2020 will lead to inadequate physical activity and consequently increased risk of cardiovascular disease worldwide [15], this review will also explore the current landscape and future possibilities of home-based, unsupervised exercise interventions.

## Methods

The Preferred Reporting Items for Systematic Reviews and Meta-Analyses (PRISMA) checklist was used for this review [16], and was registered with the international database of prospectively registered systematic reviews in health and social care (PROSPERO Registration Number: CRD42020221206).

### Information sources and search strategy

Electronic databases (PubMed, Cochran Library, Web of Science, and Igaku Chuo Zasshi) were searched for studies published using all available records up to May 31, 2020. The search expression used was as follows;

"breast cancer"[Title/Abstract] AND ("high intensity interval"[Title/Abstract] OR "high intensity intermittent"[Title/Abstract] OR "aerobic interval"[Title/Abstract]) AND (exercise OR training) AND (randomized controlled trial[pt] OR controlled clinical trial[pt] OR randomized[tiab] OR placebo[tiab] OR randomly[tiab] OR trial[tiab] OR groups[tiab] NOT (animals [mh] NOT humans [mh]))

All studies with keywords related to HIIT interventions for breast cancer survivors were included.

### Inclusion criteria

Inclusion criteria were studies published in English and Japanese (only those with full text available) that included HIIT in the intervention and were conducted in breast cancer survivors. HIIT was defined as exercise consisting of multiple repetitions of short bursts (≤4 min) of high-intensity (≥90% of maximal oxygen uptake [VO_2_max], peak oxygen uptake [VO_2_peak] or rating of perceived exertion [RPE] ≥ 18) aerobic exercise (e.g., running or cycling) alternated with low-intensity exercise or passive rest. Studies of interventions that combined HIIT with resistance training or aerobic training were also included in the review. When multiple datasets were available from the same research group or follow-up data were available for the same cohort of participants, the earliest published dataset was used.

### Study selection and data extraction

Irrelevant articles were excluded from the review by screening the titles and abstracts displayed in the search results (KT). Next, methods of intervention (exercise duration/frequency, exercise intensity, mode of exercise, HIIT intervals, and intervention setting) and outcomes (cardiorespiratory fitness, muscle strength, indicators of cardiotoxicity/cardiovascular function, health-related quality of life [HRQOL], fatigue, related biomarkers, adverse events, and compliance) were determined by reviewing the full text. The full text was independently reviewed by two of the authors (KT and EO). These outcomes were selected to investigate the effects of HIIT on physical function as the primary outcome of interest, as well as the effects of HIIT on areas of clinical concern for breast cancer survivors (HRQOL, fatigue, and cardiotoxicity/cardiovascular function) and safety of and compliance with HIIT among breast cancer survivors.

### Risk of bias assessment

The Cochrane risk of bias tool was used to maintain internal validity [17]. All of the authors (KT, YM, EO) assessed selection bias, performance bias, detection bias, attrition bias, reporting bias, and other biases. The aim of this review is not assess the effect of HIIT on clinical outcome in breast cancer survivors, however, publication bias may affect the number of studies published. Any disagreements between reviewers were solved through discussion on a video conference.

## Results

### Search outcome

**Fig. 1:**
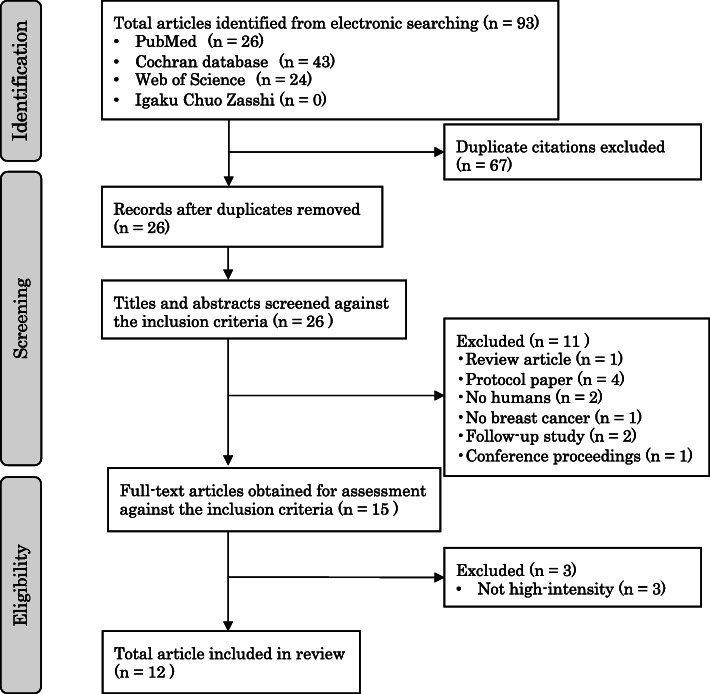
PRISMA flow diagram

A total of 93 search results were obtained from the four databases, but 26 were duplicates and were therefore excluded. After screening, 9 studies were excluded from the review based on their title and abstract, and 2 more studies were excluded because they were follow-up studies of the same cohort. All 15 articles extracted were available in full text. After the full text of the remaining studies was carefully reviewed, an additional 3 studies were excluded for not meeting the exercise intensity criteria described in the Methods section. Finally, a total of 12 studies satisfied the inclusion criteria (Fig. 1).

Tables 1 and 2 summarize the studies included in the review. Table 1 lists interventions conducted during breast cancer treatment, and Table 2 lists interventions conducted after initial cancer treatment. Each table lists the authors, sample size, a summary of the HIIT program, outcomes, whether the intervention was supervised or unsupervised, and the intervention setting (lab-based, community-based, or home-based) for each study. Summaries of the HIIT programs include the duration of training, frequency, mode of exercise, intensity, and intervals.

**Table 1:** Characteristics of the included studies with intervention during treatment

Study	Sample size	HIIT				Supervised OR Unsupervised	Lab-based OR Home and community-based
		Duration and frequency (total times)	Mode	Intensity	Interval and recovery durations		
Lee et al. (2019a,b, 2020) [18–20]	30	8 weeks, 3×/week (24)	Cycle ergometer	90% PPO	7 × 1 min cycling, 2 min active recovery, total 19 min	Supervised by exercise trainer	Lab-based
Mijwel et al. (2020) [21]	182	16 weeks, 2×/week (32)	Cycle ergometer	16–18 RPE	3 × 3 min cycling, 1 min passive recovery, total 11 min	Supervised by exercise physiologist or oncology nurse	Lab-based
Mijwel et al. (2018) [22]	23	16 weeks, 2×/week (32)	Cycle ergometer	16–18 RPE	3 × 3 min cycling, 1 min passive recovery, total 11 min	Supervised by exercise physiologist or oncology nurse	Lab-based
Mijwel et al. (2018a,b) [23, 24]	206	16 weeks, 2×/week (32)	Cycle ergometer	16–18 RPE	3 × 3 min cycling, 1 min passive recovery, total 11 min	Supervised by exercise physiologist or oncology nurse	Lab-based
Schulz et al. (2018) [25]	16	6 weeks, 2×/week (12)	Cycle ergometer	85–100%VO_2_peak	10 × 1 min cycling, 1 min load-less recovery, total 19 min	Supervised by professional	Lab-based

**Table 2:** Characteristics of the included studies with intervention after treatment

Study	Sample size	HIIT				Supervised or Unsupervised	Lab-based or Home and community-based
		Duration and frequency	Mode	Intensity	Interval and recovery durations		
Alizadeh AM et al. (2019) [26]	52	12 weeks, 3×/week (36)	Treadmill	90–95% HRmax	4 × 4 min running, 3 min passive recovery, total 25 min	Supervised by exercise physiologist	Lab-based
Alizadeh S et al. (2019) [27]	80	12 weeks, 3×/week (36)	Treadmill	90–95% HRmax	4 × 4 min running, 3 min passive recovery, total 25 min	Supervised by exercise physiologist	Lab-based
Northey et al. (2019) [28]	17	12 weeks, 3×/week (36)	Cycle ergometer	Maximal effort	4 × 30 s, 2 min rest, total 10 min	Supervised, supervisor not described	Lab-based
Dolan et al. (2016) [14]	33	6 weeks, 3×/week (18)	Treadmill	Initial: 65% VO_2_peak, Interval 50% VO_2_peakLast: 95% VO_2_peak, Interval: < 60% VO_2_peak	First week: 4–6 × 4 min, 3 min interval, total 25–39 minlast week: 4–6 × 2 min, 2 min interval, total 25–39 min	Supervised, supervisor not described	Lab-based

### Risk of bias assessment

**Fig. 2:**
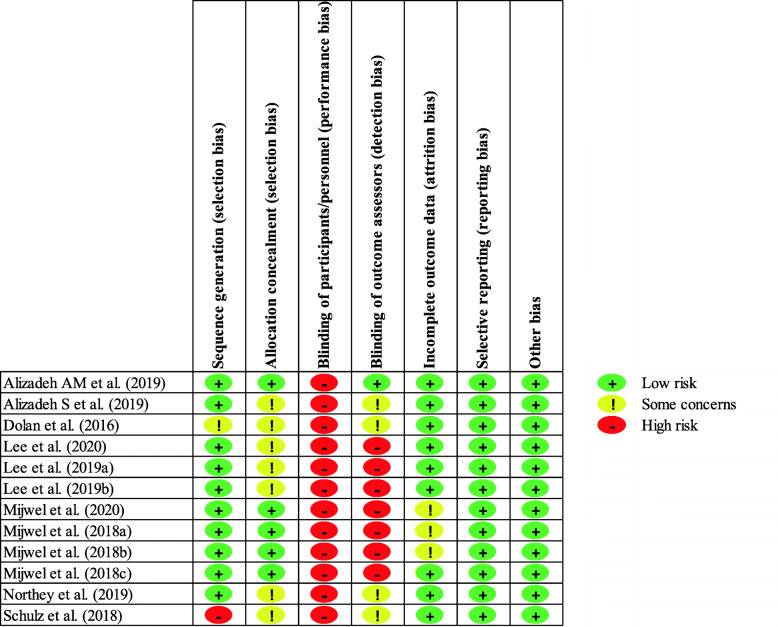
Summary of the Cochrane risk of bias tool

The results of the methodological quality assessment of the studies included in this review are summarized in Fig. 2. The proper procedure for randomly generated sequences has been fully described in ten studies [18–24, 26–28], five of which hid the assignments [21–24, 27]. Performance bias was found in all included trials. Blinding of participants is not possible due to the characteristics of exercise interventions. However, these do not pose a threat to internal validity. Only one study [27] blinded outcome assessors. Three trials found a high risk of having incomplete outcome data [21–23].

### Intervention timing (during or after treatment)

Eight studies involved interventions during breast cancer treatment and 5 involved interventions after treatment. Of the 8 interventions conducted during treatment, 3 were conducted by Lee et al., 4 by Mijwel et al., and 1 by Schulz et al. The intervention was started at the same time as chemotherapy in 7 of those 8 studies. In the remaining study, the participants received chemotherapy before and during the intervention. Two of the studies of interventions conducted after treatment revealed the timing of the intervention: participants in the studies by Alizadeh AM et al. and Alizadeh S et al. started the intervention no earlier than 1 month after completing chemotherapy and/or radiotherapy. The studies by Northey et al., and Dolan et al. did not specify the timing of the intervention.

### Setting (lab-based HIIT or community-based HIIT)

All of the 12 studies investigated lab-based interventions. All of the lab-based interventions conducted during breast cancer treatment used a cycle ergometer. Three of the interventions conducted after treatment used a treadmill, and one used a cycle ergometer.

### Exercise supervision

No studies of unsupervised HIIT have been conducted to date, and thus all the studies in this review investigated supervised interventions. Two of the 12 studies did not specify who supervised the intervention. In those that did specify, the supervisor was an exercise trainer [18–20], an exercise physiologist [21–24, 26, 27], or an oncology nurse [21–24]. In Mijwel et al., the intervention was supervised by an exercise physiologist or oncology nurse. In Schulz et al., the intervention was supervised by a professional, but no further details were provided [25].

### Exercise training protocols

Studies were sorted by HIIT protocol. The 12 studies included multiple studies conducted by the same research groups. In the group of studies on interventions during breast cancer treatment, Lee et al. (3 of 13 studies) had participants perform 7 sets consisting of 1 min of exercise at 90% peak power output determined by cardiopulmonary exercise testing, followed by 2 min of active rest, repeated 3 times per week for 8 weeks [18–20]. Mijwel et al. (4 of 13 studies) had participants perform 3 sets consisting of 3 min of exercise at RPE of 16 to 18 followed by 1 min of passive rest, repeated twice weekly for 16 weeks [21–24]. They also had participants perform resistance training (RT-HIIT group) or aerobic training (AT-HIIT group) 3 times a week on the days they did not perform HIIT. Resistance training consisted of 2 or 3 sets of 12 repetitions of resistance training exercises for 9 different muscle groups at 70 to 80% of their one-repetition maximum (1RM). Aerobic training consisted of 20 min of cycling at an RPE of 13 to 15. Schulz et al. had participants perform HIIT and resistance training as a group twice weekly for 6 weeks [25]. For HIIT, participants performed 3 sets consisting of 3 min of exercise on a cycle ergometer at an intensity of 85 to 100% VO_2_max followed by 1 min of active rest. For resistance training, they performed 8 to 12 repetitions of resistance training exercises for major muscle groups at 60 to 80% 1RM.

In the group of studies involving interventions after initial breast cancer treatment, Alizadeh AM et al. and Alizadeh S had participants perform 4 sets consisting of 4 min of inclined running at an intensity of 90 to 95% HRmax followed by 3 min of passive rest, repeated 3 times weekly for 12 weeks. Northey et al. had participants perform 4 sets consisting of 30 s of maximum-intensity pedaling followed by 2 min of rest, repeated 3 times weekly for 12 weeks [28]. Dolan et al. had participants perform an HIIT program on a treadmill that involved incrementally increasing exercise intensity over the intervention period in 3 weekly sessions for 6 weeks. The intervention started with 4 to 6 sets of 4-min running at 65% VO_2_max at 3-min intervals (50% VO_2_peak), but the intensity was increased to 90% VO_2_peak at the 13th session in Week 5, and ultimately to 4 to 6 sets of 2-min running at 95% VO_2_peak at 2-min intervals (< 60% VO_2_peak) in the final week (Week 6) [14].

### Outcomes

Studies were also grouped by outcomes (cardiorespiratory fitness, muscle strength, indicators of cardiotoxicity/cardiovascular function, HRQOL, fatigue, related biomarkers, adverse events, and compliance). Five studies evaluated cardiorespiratory fitness, all using VO_2_peak. Three studies evaluated muscle strength, 2 using 1RM and 1 using maximum isometric contraction. One study evaluated cardiovascular function, and used endothelial function in terms of brachial artery flow mediated dilation (baFMD) and carotid intima-media thickness (cIMT) as an indicator. One study evaluated HRQOL, and used the European Organization for Research and Treatment of Cancer Quality of Life Questionnaire-Core 30 (EORTC QLQ-C30) [22]. One study evaluated fatigue, one using the 22-item Piper Fatigue Scale (PFS) [22].

Twelve studies evaluated HIIT compliance. The compliance rates in each study were as follows. Lee et al. reported an HIIT compliance rate of 82.3% across their studies. Mijwell et al. reported a compliance rate of 80 to 83% for HIIT plus resistance training and 57 to 75% for HIIT plus aerobic training across their studies. Schulz et al. and Alizadeh AM et al. reported HIIT compliance rates of 97 and 85%, respectively. Northey et al. reported an HIIT compliance rate of 78.7% across their studies. Dolan et al. reported a compliance rate of 99% for aerobic interval training. None of the studies that evaluated adverse events associated with HIIT reported any such events.

## Discussion

HIIT interventions during breast cancer treatment have been aimed at preventing or reducing cardiovascular effects of chemotherapy since Schulz et al. first investigated the feasibility of such interventions in 2018. HIIT interventions for breast cancer survivors have been investigated in only 3 studies since Dolan et al. published their study in 2016, and these studies have only investigated a narrow range of outcomes such as safety and cardiorespiratory fitness. Especially, there is no evidence to validate the efficacy of home-based HIIT, and future research results are awaited. The main findings of this review are that all HIIT interventions for breast cancer survivors to date were supervised, and nearly all were lab-based. Breast cancer survivors may face several challenges when trying to start exercising, including that resistance training and aerobic training are time-consuming, that gym memberships and exercise classes are expensive, and that access to exercise facilities may depend on where they live. In fact, the top responses in a survey that asked breast cancer survivors about barriers to exercising were lack of time and lack of access to facilities [29]. Therefore, home-based HIIT programs for breast cancer survivors will be necessary to overcome these barriers. In the following sections, individual aspects of the reviewed studies are discussed.

### Setting of HIIT

All past studies of interventions during and after breast cancer treatment were lab-based. Possible reasons for this include that the purpose of these studies was to evaluate safety or feasibility, and that exercise intensity was exactly defined to validate the efficacy of HIIT. All past studies regardless timing of intervention were also lab-based, while there is no study of home-based or community-based. Recent review paper has shown that home-based exercise is an effective method for promoting exercise in cancer survivors [30]. Future studies will need to determine how to assist people in engaging in home-based or community-based HIIT exercise programs.

### Exercise supervision

In all past studies of interventions during breast cancer treatment, the intervention was supervised by an exercise professionals or oncology nurse. In all past studies of interventions after breast cancer treatment, the intervention was also supervised by an exercise professional. A study comparing supervised and unsupervised HIIT interventions in healthy adults [31] showed that supervised interventions produced greater improvements in cardiorespiratory fitness, but unsupervised interventions still produced significant improvements. Another study of unsupervised HIIT in which participants exercised alone also showed improvements in cardiorespiratory fitness [32].

### Exercise training protocols

The following subsections discuss about frequency and period, type of exercise, intensity, and exercise and recovery intervals in the studies reviewed.

#### Frequency and intervention period

The period of HIIT interventions during breast cancer chemotherapy ranged from 6 [25] to 16 weeks [21–24]. The 16-week intervention was a combined intervention with resistance training or aerobic training. The longest HIIT-only interventions were 8 weeks [18–20]. The frequency of sessions during the intervention period was 3 times per week in 3 studies and twice per week in 5 studies. The smallest total number of sessions was 12, and the largest was 36.

The period of HIIT interventions for survivors in studies reviewed in this article ranged from 6 to 12 weeks. The frequency of sessions during the intervention period was 3 times per week in all 4 studies. The smallest total number of sessions was 18, and the largest was 36. In a study investigating the frequency and period of interval training programs, Edward Fox found that a 7-week HIIT program conducted 2 days per week produced comparable improvement in VO_2_max to a 7- or 13-week HIIT program conducted 4 days per week [33]. The study also found that training 2 days a week produced comparable improvement in cardiorespiratory fitness to training 4 days a week, and other studies reviewed in the present article also showed that a frequency of 2 to 3 times per week improves cardiorespiratory fitness [33]. Based on this evidence, a frequency of 2 to 3 times per week can be considered appropriate for HIIT interventions for breast cancer survivors. In this review, a significant improvement in cardiorespiratory function was found in the 6-week study, which was the shortest intervention period [14]. Therefore, it can be concluded that an intervention period of at least 6 weeks is necessary for HIIT to be effective.

#### Type of exercise

The mode of training was exercise on a cycle ergometer in all studies of interventions during cancer treatment. These studies likely selected a cycle ergometer because they decided to use VO_2_max as an indicator of exercise intensity during training in order to evaluate safety and feasibility of HIIT during breast cancer treatment, and a cycle ergometer allows for quantification of work. The mode of training in studies in cancer survivors was a treadmill in 3 studies [14, 26, 27] and cycling in 1 study [28].Almost all past studies of HIIT in subjects other than breast cancer survivors used equipment that allows for quantification of work (e.g., a cycle ergometer or treadmill) because VO_2_max was set as the indicator of exercise intensity. Exercise intensity is the most important factor in HIIT, and thus it is ideal to be able to quantify work. However, this requires exercise equipment, which makes such programs unfeasible for widespread implementation.

#### Intensity

In all of the studies of interventions during treatment, the relative exercise intensity set at the start of the intervention was maintained until the end of the intervention, which would have resulted in the absolute intensity increasing over the duration of training. It is best to use a physiological index to calculate exercise intensity during HIIT, but Mijwel et al. used a rating of perceived exertion of 16 to 18 in their study. Past studies of home-based HIIT interventions that used the “talk test” (intensity should be great enough that talking is difficult) [32] or a modified Borg scale score of 6 to 8 (“very hard”) [34] as an indicator of exercise intensity showed significant improvement in the primary endpoint of cardiorespiratory fitness. Therefore, even though Mijwel et al. may have used a slightly lower or unclear exercise intensity for HIIT compared with other studies, that intensity may have been sufficient to increase VO_2_max.

In studies of interventions in survivors, the relative exercise intensity set at the start of the intervention was maintained until the end of the intervention in 3 of 4 studies, and the relative exercise intensity was increased incrementally from the start of the intervention in 1 study. Northey et al. [28] had participants pedal at maximum intensity for 30 s, which was likely the most intense burst of exercise out of all 4 studies (and also including interventions during cancer treatment).

#### Interval and recovery durations

In studies of interventions during treatment, the exercise and recovery intervals differed greatly depending on the HIIT exercise intensity. In the HIIT programs investigated in these studies, the exercise interval ranged from 1 to 3 min, the recovery interval from 1 to 2 min, the number of sets from 3 to 10, and the total exercise duration from 11 to 19 min.

In the HIIT programs used in studies of cancer survivors, the exercise interval ranged from 30 s to 4 min, the recovery interval from 2 to 3 min, number of sets from 4 to 6, and total exercise duration from 10 to 39 min. HIIT is currently attracting global interest, and there is ongoing debate about its methodology. As such, the optimal exercise interval, recovery interval, and number of sets have not yet been established, and studies on HIIT should consider these aspects alongside exercise intensity and feasibility. High intensity is most important to maximize the effects of HIIT. Northey et al., whose intervention used the most intense bursts of exercise of any study included in this review, had participants perform 4 sets consisting of 30 s of maximum-intensity pedaling followed by 2 min of rest. This method is similar to ones used for the healthy general population and athletes [35]. This indicates that exercise and recovery intervals in HIIT for breast cancer survivors can be investigated using methods similar to HIIT for the healthy general population. It will be necessary to develop a program with the most efficient exercise and recovery intervals optimized for breast cancer survivors on the basis of findings from studies on HIIT conducted to date.

### Outcomes

#### Cardiorespiratory fitness

Of the 3 studies of interventions during treatment that evaluated cardiorespiratory fitness, 1 found that the HIIT intervention significantly increased cardiorespiratory fitness, and 2 found no difference. However, the 2 studies that found no difference did find that cardiorespiratory fitness decreased significantly at the end of the study in the control group, indicating that HIIT does prevent the reduction in cardiorespiratory fitness by cancer treatment. Of the 2 studies of interventions for cancer survivors that evaluated cardiorespiratory fitness, 2 found that the HIIT intervention significantly increased cardiorespiratory fitness. These findings suggest that HIIT has the effectiveness for improving cardiorespiratory fitness in breast cancer survivors.

#### Muscle strength and muscle mass

Both of the studies of interventions during cancer treatment that evaluated muscle strength showed significant improvements. Mijwell et al. and Schulz et al., who investigated interventions during breast cancer treatment, combined HIIT with resistance training. Mijwell et al. used back muscle strength (measured by isometric contraction) and grip strength to evaluate muscle strength. Schulz et al. used leg press 1RM to evaluate muscle strength. The effect of HIIT alone on muscle strength is not clear from these studies because resistance training had a strong effect. Mijwell et al. also evaluated muscle cross-sectional area (CSA) after the HIIT intervention. In that study, although it is unclear to what degree HIIT contributed to this result, they found that CSA of type II muscle fibers increased significantly and satellite cells increased after their HIIT plus resistance training intervention. Only 1 study of survivors evaluated muscle strength and found a significant increase. Dolan et al. used an intervention consisting solely of aerobic interval training and evaluated muscle strength by leg press 1RM. One study found that lower body muscle strength in breast cancer survivors is lower than or comparable to that in the general population [36], and HIIT has been shown to increase lower body muscle mass in healthy young men [37]. Although further evidence is necessary, HIIT shows promise for increasing muscle strength in breast cancer survivors, a population with reduced muscle strength deficit.

#### Cardiotoxicity and cardiovascular function

Two studies of interventions during treatment, both by Lee et al., evaluated the effects of HIIT on cardiotoxicity and vascular endothelial function. Breast cancer chemotherapy can be cardiotoxic, reduce cardiopulmonary function, and damage cardiac muscle tissue. Moderate-intensity exercise interventions added to chemotherapy have been investigated as a means to address these issues, and systematic reviews have shown the efficacy of such interventions [38, 39]. However, the authors noted that research on exercise interventions to reduce cardiotoxicity is still initial stage, and further research into aspects such as intervention timing and intensity is necessary. Based on the findings of this review, Lee et al. conducted an HIIT program aimed at improving vascular endothelial function in patients undergoing chemotherapy for breast cancer. They found promising evidence that HIIT may reduce cardiotoxicity, including improvements in vascular endothelial function and cardiovascular biomarkers. Further research into the efficacy of high-intensity exercise such as HIIT for reducing cardiotoxicity is necessary to confirm its suitability in cancer survivors.

#### HRQOL, fatigue, and biomarkers

HIIT shows great potential for improving measures of physical function such as cardiorespiratory fitness and muscle strength. However, research on its effects on HRQOL and fatigue is lacking. Only 1 study evaluated HRQOL, and showed that HIIT improved HRQOL [22]. Only 1 study evaluated the effects of HIIT on fatigue. Mijwel et al., who investigated combination of HIIT plus resistance training or aerobic training, observed no change in fatigue evaluated by the PFS [22]. Alizadeh AM et al. found that HIIT significantly reduced levels of interleukin (IL)-6 [26]. In summary, there is insufficient evidence regarding the effects of HIIT on HRQOL and fatigue. Further research should be conducted to determine the efficacy of HIIT for these outcomes in breast cancer survivors.

#### Compliance rate and adverse events

All 8 studies of HIIT interventions during treatment reported compliance rates, and those rates ranged from 57 to 97%. The compliance rate was 82.3% for HIIT alone, 80 to 97% for HIIT plus resistance training, and 57 to 75% for HIIT plus aerobic training. Six of the 8 studies during chemotherapy reported about adverse events and all 6 reported no adverse events, thus demonstrating that prescription of HIIT is extremely safe. Four of the 5 studies of HIIT interventions after treatment reported compliance rates, which ranged from 78.7 to 99%. Two of the 4 studies evaluated adverse events, and all 2 reported no adverse events, thus demonstrating that prescription of HIIT for breast cancer survivors is also safe.

### Perspective

#### Efficacy of HIIT in cancer survivors

The effects of HIIT on cardiorespiratory fitness were confirmed and comparable between interventions conducted during treatment (significant increase in 1 study, amelioration of treatment-related reduction in 2 studies) and after treatment (significant increase in 2 studies). HIIT compliance rates and incidence of adverse events also showed similar trends between interventions conducted during and after treatment, thus demonstrating the promising efficacy of HIIT. However, few studies examined muscle strength and mass or changes in cardiotoxicity or cardiovascular function after HIIT intervention in survivors. Therefore, further research on these outcomes is necessary. One of the limitations of this review is that the overall number of studies included was small. Also, this review did not focus on outcome differences. However, in assessing the risk of bias, the overall risk of bias is considered to be low, except for the higher risk of blindness due to the specificity of the exercise intervention.

#### Possibilities for home-based HIIT

A wide variety of basic and applied research has investigated HIIT in the general population. Given that HIIT is already known to improve cardiorespiratory fitness, more recent studies have investigated the feasibility of HIIT programs without specialized equipment or supervision. Blackwell et al. compared the effects of unsupervised bodyweight HIIT (home HIIT) and supervised HIIT using a treadmill (lab HIIT) on VO_2_max. They found that both lab HIIT (pre 26.50 ± 6.31, post 31.00 ± 6.69 mL/kg/min, *p* < 0.001) and home HIIT (pre 27.77 ± 4.75, post 29.98 ± 6.09 mL/kg/min, *p* < 0.05) significantly improved VO_2_max, but lab HIIT produced a significantly greater increase than home HIIT (p < 0.05) [31]. In contrast, Menz et al. found that home HIIT (pre 49.5 ± 6.6, post 54.4 ± 5.3 mL/kg/min, p < 0.001) produced comparable improvement in VO_2_max to lab HIIT(pre 47.8 ± 5.6, post 54.1 ± 5.6 mL/kg/min, p < 0.001) [40]. A systematic review of bodyweight HIIT methodology has also been conducted [41]. The findings of these studies suggest that bodyweight HIIT is beneficial for increasing cardiorespiratory fitness, and a home-based bodyweight HIIT program should be developed for breast cancer survivors. Home-based HIIT for breast cancer survivors has only ever been investigated in 1 study protocol [42]. In that study, participants performed bodyweight HIIT exercises at home, and their exercise was monitored with a wearable device [42].

## Summary

All studies on HIIT for breast cancer survivors used lab-based, supervised interventions, while none of the home-based HIIT have reported so far. HIIT is a time-efficient method for increasing cardiorespiratory fitness in breast cancer survivors, but further research is necessary to determine its effects on other outcomes such as HRQOL, fatigue, muscle function, and cardiovascular function because few studies have evaluated those outcomes. Due to the lack of evidence of benefit from home-based HIIT for breast cancer survivors, additional studies should be conducted to confirm the effects of such programs.

## Data Availability

Not applicable.

## Abbreviations

HIIT: High-intensity interval training
PRISMA: Preferred reporting items for systematic reviews and meta-analyses
PROSPERO: International prospective register of systematic reviews
VO_2_max: Maximal oxygen uptake
VO_2_peak: Peak oxygen uptake
RPE: Rating of perceived exertion
HRQOL: Health-related quality of life
RT: Resistance training
AT: Aerobic training
1RM: One-repetition maximum
baFMD: Brachial artery flow mediated dilation
cIMT: Carotid intima-media thickness
EORTC QLQ-C30: european organization for research and treatment of cancer quality of life questionnaire-core 30
PFS: Piper fatigue scale
CSA: Cross-sectional area
IL: Interleukin
